# Fantastic niches and where to find them: the current diagnosis and management of uterine niche

**DOI:** 10.52054/FVVO.14.1.003

**Published:** 2022-04-03

**Authors:** S McGowan, C Goumalatsou, A Kent

**Affiliations:** Gynaecology Department, Royal Surrey County Hospital, Royal Surrey NHS Foundation Trust, Egerton Road, Guildford, Surrey, United Kingdom; Women’s and Children’s Department, St Richard’s Hospital, Spitalfields Road, Chichester, United Kingdom; Gynaecology Department, Royal Surrey County Hospital, Royal Surrey NHS Foundation Trust, Egerton Road, Guildford, Surrey, United Kingdom

**Keywords:** Niche, isthmocele, caesarean scar defect, sacculation

## Abstract

**Background:**

Caesarean section (CS) scar niche is a well recognised complication of caesarean delivery and is defined as an indentation at the site of the CS scar with a depth of at least 2mm.

**Objectives:**

To review systematically the medical literature regarding the current diagnosis and management of uterine niche

**Materials and methods:**

We carried out a systematic review using MeSH terms ‘niche’ OR ‘sacculation’ OR ‘caesarean scar defect’ OR ‘caesarean section scar’ OR ‘uterine defect’ OR ‘isthmocele.’ Articles included were peer-reviewed and in English language.

**Main outcome measures:**

Prevalence, symptoms, diagnosis, pathophysiology and management of uterine niche.

**Results:**

CS scar niche is common and, in a subgroup, produces a range of symptoms including post-menstrual bleeding, dyspareunia and subfertility. It may be linked to use of locked sutures during CS closure. Niche repair can be achieved laparoscopically or hysteroscopically and appears to improve symptoms, although solid conclusions regarding fertility outcomes cannot be drawn.

**Conclusions:**

CS scar niche is associated with a range of symptoms. Repair may aid subfertile patients and those with post-menstrual spotting. The presence of a niche is probably irrelevant in the absence of symptoms.

**What is new?:**

LNG-IUS and surgical repair appear to improve symptoms in those with a niche.

## Introduction

The incidence of caesarean section (CS) is broadly rising worldwide and in the UK is currently at 29% (Maternity Services Monthly Statistics, December 2020). Whilst CS has well established obstetric complications, increasing attention is drawn to the long-term complications arising in the non-pregnant population including that of a uterine niche.

A uterine niche is a reservoir-like pouch in the anterior uterine isthmus located at the site of a previous CS scar (Gubbini et al., 2008). A wedge-shaped defect was first described using hysterosalpingography (HSG) by Poidevin (1961). On ultrasound it may be defined as an anechoic or hypoechoic structure at the site of the previous CS (Thurmond et al., 1999; Monteagudo et al., 2001; Bij de Vaate et al., 2011; Naji et al., 2012). Monteagudo et al. (2001) introduced the term “niche” in 2001, but the condition has also been termed a sacculation, isthmocele, a caesarean/post- caesarean scar defect, scar pouch and diverticulum. It has been postulated that such niches could be a source of gynaecological symptoms (Thurmond et al., 1999) and the term ‘sacculation syndrome’ has been suggested linking CS with abnormal bleeding and the sacculation or niche by Kent and Waters (2009).

In 2019 a European Taskforce published a document using the term “uterine niche;” they defined a niche as an indentation at the site of the CS scar with a depth of at least 2mm. There were 3 subclassifications 1) simple niche 2) simple niche with one branch 3) complex niche (>1 branch, where a branch is a thinner part of the main niche, directed towards the serosa with a width measuring less than the main niche) (Jordans et al., 2019). The rise in CS rates have brought increased levels of long-term gynaecological morbidity and the niche is thought to be responsible for symptoms as varied as post- menstrual spotting, pelvic pain and dyspareunia.

## Materials and methods

**Figure 1:**
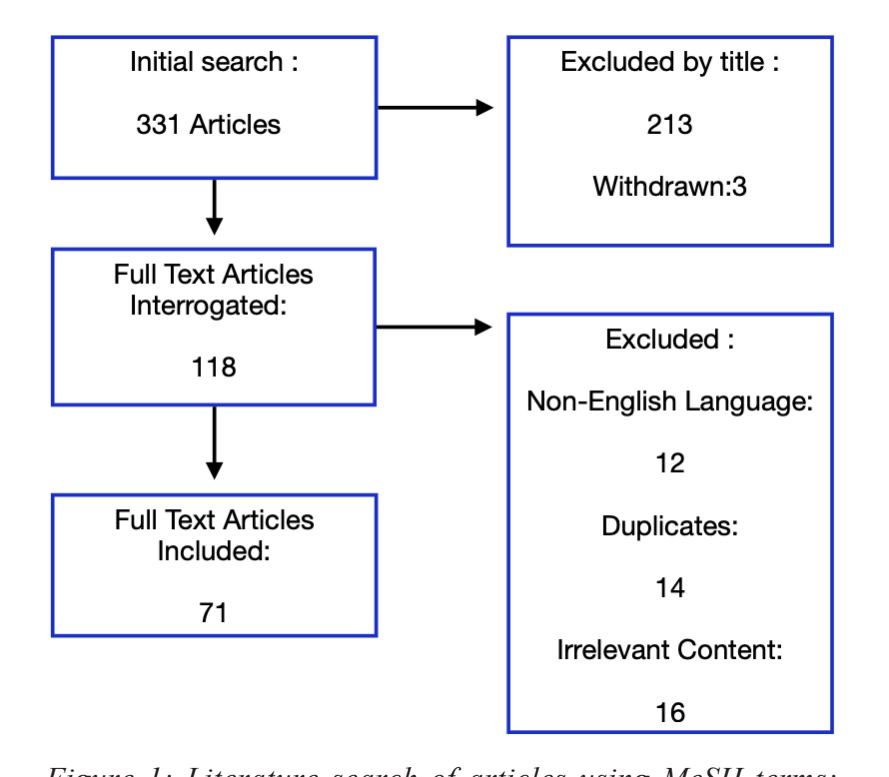
Literature search of articles using MeSH terms; ‘Niche’ OR ‘sacculation’ OR ‘caesarean scar defect’ OR ‘caesarean section scar’ OR ‘uterine defect’ OR ‘isthmocele.’ Articles were included which were English language and peer-reviewed, published between 1990 and 2021.

We carried out an extensive search to gain a broad overview of the prevalence, symptoms, diagnosis, pathophysiology and management of uterine niche, using both Google Scholar and PubMed. The following MeSH terms were included: ‘Niche’ OR ‘sacculation’ OR ‘caesarean scar defect’ OR ‘caesarean section scar’ OR ‘uterine defect’ OR ‘isthmocele.’ Articles were included which were English language and peer-reviewed, published between 1990 and 2021. Two authors independently selected abstracts and subsequently full text of appropriate articles, inspecting reference lists for inclusion of further articles for review. Figure 1 demonstrates our literature search.

### Prevalence

While it appears reasonable to expect the prevalence of niche to be rising mirroring that of CS, the exact prevalence is difficult to determine.

This reflects variations in population, the definition and criteria for niche diagnosis and the detection method used. They do however appear to occur frequently after CS. Within Bij De Vaate et al’s systematic review, prevalence detected by transvaginal ultrasound (TVUS) varied between 24-70% in a random population with a previous history of CS. This rose to 56-84% within a random population when sonohysterography (SHG) was used (Bij de Vaate et al., 2014). In the studies that met the STROBE criteria; Osser et al. (2010) showed a prevalence of 70% using TVUS and 84% using SHG where the niche was defined as any indentation or defect in the scar while Bij de Vaate et al. (2011) found a prevalence of 24% using TVUS and 56% using SHG where the niche was defined as an anechoic area at the scar site with a depth of at least 2mm. The prevalence has been shown to be higher in symptomatic women. Van der Voet et al. (2014 a) compared TVUS with gel instillation sonohysterography (GIS) at 6-12 weeks following CS. Detection via GIS revealed a higher prevalence (49.6 vs 64.5%), increased niche depth and thinner residual myometrium. Large defects appear less frequent with prevalence between 11-45% dependent on the definition used to describe the niche size (depth of 50-80% of the myometrium, or residual myometrium ≤2.2mm) (Bij de Vaate A et al., 2014).

### Pathology

In 1995 Morris published a paper noting a number of pathological changes within the CS scar. His sample of 51 hysterectomy specimens were from women who suffered from abnormal uterine bleeding unresponsive to medical treatment who had previously had a CS. In over 90% there was residual suture material with foreign body giant cell reaction. In 75% of specimens there was a distortion and widening of the lower uterine segment. In 65% there was moderate to marked lymphocytic infiltration and capillary dilatation. In 61% an “overhang” of endometrium above the scar tissue was described, noted to be congested. In 59% red cells were seen within the endometrial storm of the scar suggesting recent haemorrhage. Fragmentation and breakdown of the endometrium within the scar was noted in 37%, iatrogenic adenomyosis in 28% and polyp formation conforming to scar recess contours in 16% (Morris, 1995). The histological features of the niche itself has had minimal attention. Most show endocervical-type mucosa that frequently shows cystic dilatation, hybrid endocervical endometrial mucosa typical of the lower uterine segment, with atypic and fibroblastic storm frequently seen (Karpathiou et al., 2020).

### Aetiology and risk factors

A number of authors have attempted to identify and categorise factors associated with niche development (Bij de Vaate et al., 2014; Vervoort et al., 2015). Vervoort et al. (2015) proposed surgery- related risk factors which may be modified, and patient-related risk factors such as those impacting wound healing, which are not easily modified.

One hypothesis is that low incisions at the time of CS disrupt mucus producing cells within cervical tissue and this impairs wound healing. This may also induce cyst formation or increase niche size by mucin disrupting approximated myometrium. Vervoort et al. (2015) noted large niches located lower in the uterus, often containing mucus, closely related to nabothian cysts. A 2-centre RCT with 122 patients looked at hysterotomy level and development of a large (≤2.5mm residual endometrium) uterine defect. Women were assessed 6-9months after delivery via SHG. Low incisions were defined as 2cm below the vesicouterine fold with high incisions 2cm above. A defect rate of 7% was found in the high incision group vs 41% in the low incision group, a 6-fold increased risk in the low incision group. There were no differences in operative complications, perinatal outcomes or subsequent pregnancy complications (Vikhareva et al., 2019). An earlier study by Vikhareva Osser (2010) also demonstrated a large niche in 53% of patients where the CS was performed at ≥5cm cervical dilatation. Osser et al. (2010) also described low incisions and cervical dilatation ≥5cm as independent risk factors for developing large niches. The timing of CS may influence the level of the scar incision which makes sense because in active labour, after the endocervix has effaced and merged with the lower segment, it is entirely possible to make an incision in the endocervix. Zimmer et al. (2004) reported a higher prevalence with CS in active labour after cervical effacement (76% vs 57%, p=0.001). Osser et al. (2010) also showed that a longer labour with a duration of ≥ 5 hours was also associated with larger defects. A recent prospective cohort study of 200 women showed an increased incidence of defects diagnosed by TVUS after emergency CS compared to elective (Dosedla et al., 2020). There appears to be inconsistency with results however, as some other studies have shown an increased risk of niche formation at less cervical dilatation, with presence of labour or emergency CS not being shown as risk factors (Yazicioglu et al., 2006; Hayakawa et al., 2006).

A further hypothesis relates to factors causing incomplete closure of the uterine wall. Vervoort et al. (2015) suggested that omission of the deeper muscular layer of the uterus may lead to myometrial disruption and niche development; they suggested non-perpendicular or tangential suturing and endometrial saving techniques as potential causes. It should be noted that uterine closure techniques vary significantly between countries. In the UK double layer closure is advised (NICE Clinical Guideline 132, 2011) while in other countries single layer closure is much more common. Vervoort et al. (2015) reported that 92% of 528 gynaecologists surveyed in the Netherlands used a single layer technique. 86% did not close the peritoneum. In a number of the studies mentioned above, for example, almost all participants had single layer closure (Bij de Vaate et al., 2014; Van der Voet et al., 2014a). Both the CORONIS and CAESAR RCTs failed to show significant differences in surgical interventions including single vs double layer closure in the short term. (Abalos et al., 2013; CAESAR study collaborative group, 2010). A Cochrane review by Dodd et al. (2014) also failed to show differences in short term outcomes; however, longer term outcomes including menstrual disorders and fertility issues were not examined. Di Spiezio Sardo et al. (2017) performed a systematic review and meta-analysis looking at the risk of a uterine defect following single vs double layer closure. This identified 9 RCTs with almost 4000 participants. They found no difference in defect incidence, rupture or dehiscence however did note that single layer closure was associated with a significantly thinner residual myometrial thickness (RMT) on ultrasound. The authors noted that the quality of summary estimates was low however, indicating that the true effect may be substantially different than that estimated. This publication supports earlier findings by Roberge et al. (2014). A more recent systematic review and meta- analysis by Stegwee et al. (2018) looked at the effect of uterine closure techniques on ultrasound findings and maternal outcomes. Again, single layer closure was associated with a significantly reduced RMT but also a reduced healing ratio (the ratio of residual myometrial thickness to adjacent endometrial thickness (AMT) and dysmenorrhoea. Reduced RMT and healing ratio were also found when a locking suture technique was used. The authors advocate double layer non-locking suture techniques for reduction in dysmenorrhoea and improved RMT and healing ratio. Exclusion of the decidua was also associated with a significant increase in niche prevalence (Stegwee et al., 2018). A previous randomised prospective study however showed an improved healing of the uterine incision when full thickness closure was used (Yazicioglu et al., 2006). The recently published, multi-centre RCT known as 2CLOSE investigated whether double layer closure is superior to single in terms of development of niche and post menstrual spotting symptoms (Stegwee et al., 2021). Approximately 30% of trial participants were unfortunately lost to follow-up and a further 15% excluded for amenorrhoea at 9 months postpartum. In the remaining participants, post-menstrual spotting was similar between both groups but niche prevalence on ultrasound was lower in the single unlocked group (68.9 vs 73.6%, p=0.03). When it comes to using a locked suture, Yasmin et al. (2011) showed increased blood loss and reduced RMT compared with unlocked techniques while Ceci et al. (2012) showed similar prevalence of CS defect at 6-12 months on ultrasound, but locking was associated with a larger niche.

A third hypothesis relates to activities that may induce adhesion formation between the CS site and the anterior abdominal wall such as infection, poor haemostasis, devascularisation and ischaemia. Vervoort et al. (2015) noted dense adhesions attached at the top of the wedge defect in the majority of cases, suggesting that scar retraction induces niche development, potentially exaggerated in retroflexed uteri. A number of studies have supported this observation with larger or wider niches more prevalent in women with retroflexed uteri (Bij de Vaate et al., 2014; Osser et al., 2009; Wang et al., 2009). It is unknown if the flexion of the uterus causes inadequate healing or if the retroflexion occurs following niche development. Some authors have investigated whether non- closure of the parietal peritoneum increases adhesion formation (Cheong et al., 2009), however a Cochrane review by Bamigboye and Hofmeyr (2014) suggests insufficient evidence of benefit to justify additional time and suture material needed for routine closure. Previous caesarean sections have been shown to increase the risk of a larger CS defect and incomplete wound healing (Wang et al., 2009, Antila-Långsjö et al., 2018). Finally, patient factors related to wound healing may be also associated. Increased maternal BMI, gestational diabetes and pre-eclampsia were associated with an increased risk of incomplete healing of the CS incision. (Osser et al., 2009, Antila-Långsjö et al., 2018). Obesity and diabetes are independent risk factors associated with poorer wound healing in the non-pregnant state.

### Clinical presentation

#### Abnormal uterine bleeding

**Figure 2:**
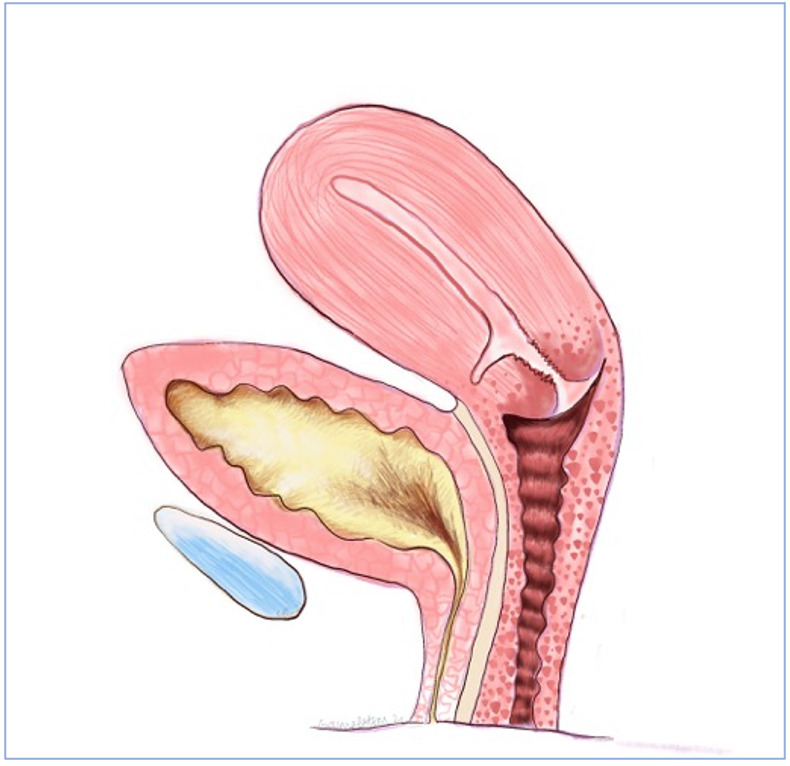
A cross section of a uterine niche.

**Figure 3:**
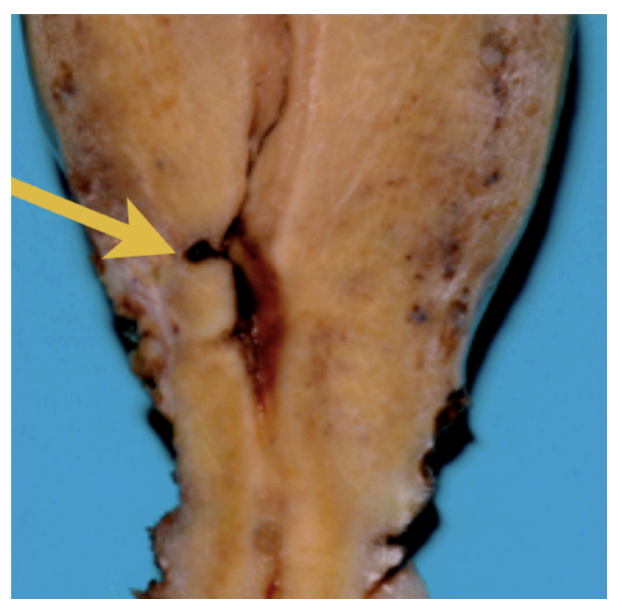
A cross section of a uterine niche from a hysterectomy specimen.

**Figure 4:**
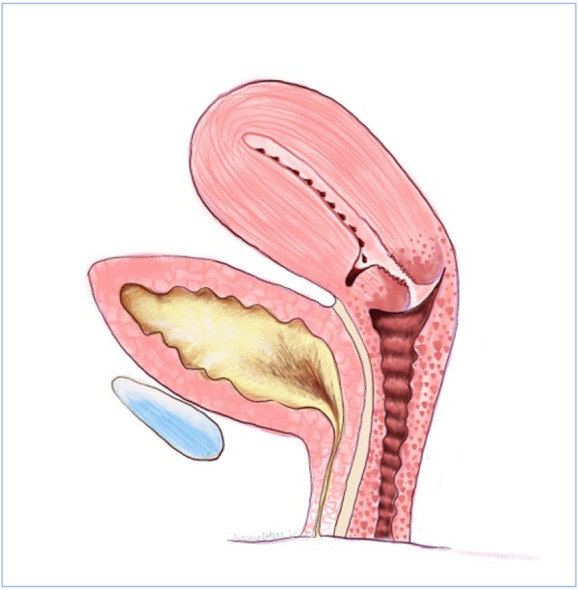
Thurmond’s proposed mechanism of symptoms due to uterine niche; where a lack of coordinated muscular contraction at the scar allows menstrual debris to collect in the defect resulting in persistent spotting after cessation of menstrual bleeding.

The most common clinical presentation of a uterine niche is with menstrual abnormalities, namely heavier periods, prolonged menstruation and post- menstrual spotting or discharge. Thurmond et al. (1999) hypothesised that lack of coordinated muscular contraction at the scar allowed menstrual debris to collect in the defect, resulting in persistent spotting after cessation of menstrual bleeding. Figures 2 and 3 show cross sections of a uterine niche while Figure 4 shows this proposed mechanism. Morris’s findings of free erythrocytes within the scar tissue associated with recent haemorrhage could suggest bleeding at the site of the defect itself (Morris, 1995). Alternatively, it has been postulated that this ongoing loss of blood-stained mucous discharge is caused by low uterine incisions disrupting mucous-producing endocervical cells. Increased prevalence of post- menstrual spotting in those diagnosed with a niche has been demonstrated in a number of studies within a random population. Antila et al. (2020) reported post-menstrual spotting in 20% of women with a niche diagnosed using SHG compared to 8.3% without a niche. This rose to 22% vs 10% when those with amenorrhoea were excluded. Bij de Vaate et al. (2011) reported post-menstrual spotting in 34% of women with niche diagnosed using SHG compared to 15% without a niche (p=0.002). When amenorrhoea was excluded this rose to 36% vs 17%. Other bleeding abnormalities were also shown to be increased within the niche group, namely postcoital bleeding, 8% vs 2% (Antila et al., 2020) and intermenstrual bleeding in 30% vs 10% (p=0.001) (Bij de Vaate et al., 2011). Van der Voet et al. (2014 a) reported post-menstrual spotting in 29% of women with a niche diagnosed with GIS within a random population compared to 7% without a niche. Women with a ratio of residual myometrium less than half of the adjacent myometrium measured by TVUS or GIS reported post-menstrual spotting symptoms more frequently. A further study, again in a random population, using TVUS and SHG did not find an association with niche presence and abnormal uterine bleeding, but did find that bleeding symptoms were more frequent in women where diverticula (term used for anechoic round structures) and cervical canal deformation at the scar site. This would appear to suggest a relationship with a CS scar and abnormal bleeding symptoms (Valenzano et al., 2006). A number of studies within a population of women with gynaecological symptoms have shown a high prevalence of bleeding symptoms in those with a niche (Thurmond et al., 1999; Wang et al 2009, Fabres et al., 2003). A larger size of niche appears to be more likely to give bleeding symptoms. Bij de Vaate et al. (2011) showed increased symptoms related to large niche volume in a random population. Within those with gynaecological symptoms, larger diameter and width niches have been shown to correlate with increased bleeding symptoms (Wang et al., 2009; Uppal et al., 2011).

#### Pelvic pain

Pelvic pain and dyspareunia associated with a niche may be related to the association of adhesions. Wang et al. (2009) identified significant pain symptoms in those found to have a niche. Dysmenorrhea was found in 53%, chronic pelvic pain in 40% and dyspareunia in 18%. It appears again that the size of the niche is relevant, with chronic pelvic pain and dysmenorrhea more likely in defects showing a greater width. Pain symptoms such as dysmenorrhea and dyspareunia have however not been shown in other studies within a random population to be associated with niche presence (Antila et al., 2020).

#### Subfertility

Subfertility has also been associated with the presence of uterine niche. Persistence of blood and mucous within the niche has been suggested to affect cervical mucus and sperm quality, obstruct sperm transport and interfere with embryo implantation (Fabres et al., 2003; Fernandez et al., 1996). Gurol-Urganci et al. (2013) showed in a meta-analysis of 1,047,644 women that CS on average reduces the probability of a subsequent pregnancy by 4%, with a likely greater effect if the CS was not elective. These studies did not evaluate for the presence of a niche. Wang et al. (2017) reported that in women with previous CS, the implantation and pregnancy rates were significantly lower than in those with a previous vaginal delivery (24.0 vs 36.7% implantation, and 40.3 vs 54.2% pregnancy, 12.5% if a niche was present, p<0.05), and difficult embryo transfer was more likely to be difficult. The reasons for this were postulated to be due to the presence of a CS scar (Wang et al., 2017). Similarly, Vissers and Sluckin (2020)found a lower implantation and live birth rate in women with a previous CS as opposed to vaginal delivery (15.9 versus 23.3% respectively [OR 0.63, 95% CI 0.45–0.87]. They found that once patients in each group had successful implantation, there was no additional impact of previous CS on ongoing pregnancy, suggesting that CS specifically inhibits implantation. (Virdtssers et al., 2020)

In early pregnancy, caesarean scar ectopic, where implantation occurs at the site of the CS scar, can have uterus- and life-threatening consequences, and if the pregnancy continues, potential for malplacentation. It is plausible that a thin RMT in combination with an anterior placenta praevia may lead to abnormally invasive placenta, although no studies have compared prevalence in those with a previous CS, with and without a niche. In terms of fetal outcomes and niche, it is also known that women who have undergone a previous full-dilatation CS are at a 6-fold increased risk of subsequent preterm birth (Levine et al., 2015) and there is an ongoing study looking into a range of factors but include presence of niche and scar position on MRI and risk of preterm birth (Carlisle et al., 2020).

While certainly uterine niche may cause significant symptoms in some patients, within the majority it remains asymptomatic. Some authors have suggested that a distinct syndrome should be defined by both the presence of the uterine niche and associated symptoms to avoid the over- treatment of an asymptomatic individual (Kent and Waters, 2009; Allornuvor et al., 2013).

### Diagnosis

A uterine niche seen as a cystic or hypoechoic distortion in the scar has been demonstrated using a number of imaging modalities. The majority of defects are wedge triangular in shape (Chen et al., 1990; Fabres et al., 2003; Osser et al., 2010). Osser et al. (2009) described 83% as this shape while Bij de Vaate et al. (2011) found 50% semicircular, 32% triangular, 10% droplet shaped and 7% were inclusion cysts.

A niche can be measured with 2D or 3D transvaginal ultrasound alone, with saline or gel contrast and with magnetic resonance imaging (MRI) (Allornuvor et al., 2013). Indeed, initially the niche was first identified using HSG in 1961 (Poidevin, 1961), transabdominal ultrasonography (TAUS) in 1982 (Burger et al., 1982) and TVUS in 1990 (Chen et al., 1990). Vaginal ultrasonography has an advantage over HSG as it allows measurement of myometrial thickness as well as accurate measurement of the defect itself. Blood or mucus accumulation may also obscure the defect when HSG is used (Florio et al., 2012). Standard TVUS is widely accessible and less invasive than HSG and ultrasound imaging using a contrast medium. TVUS will, in most cases be the initial imaging modality in those presenting with symptoms and is the most commonly reported technique in initial defect identification (Tower and Frishman, 2013).

Contrast enhanced ultrasound imaging however appears to be the current imaging standard, having higher detection rates than TVUS. A study by Antila-Langsjo et al. (2018) showed that 51% of defects diagnosed by saline contrast sonohysterography remained undiagnosed with TVUS. Defects have been noted to appear larger and with clearer margins when fluid contrast imaging methods have been used (Osser et al., 2010; Bij de Vaate et al., 2010). It is unclear if these methods show a truer assessment of the niche defect or if the pressure of the fluid exaggerates the size (Naji et al., 2012). The European Niche taskforce has attempted to provide consensus on how a niche should be assessed by ultrasound. They recommend that ultrasound is used to record the niche length, depth, RMT and AMT within the sagittal plane, and width within the transverse plane. Branches should be reported. The taskforce suggested that saline or gel infusion is preferred however has no additional value if intracavity fluid is present. Fluid is more likely to be present if the ultrasound is performed between day 7-14 of the cycle, obviating the need for more invasive terilization n of the uterine cavity. Variable pressure and use of colour flow Doppler may be useful in assessing defects. Advantages and disadvantages of 3D imaging were not considered (Jordans et al., 2019).

**Figure 5:**
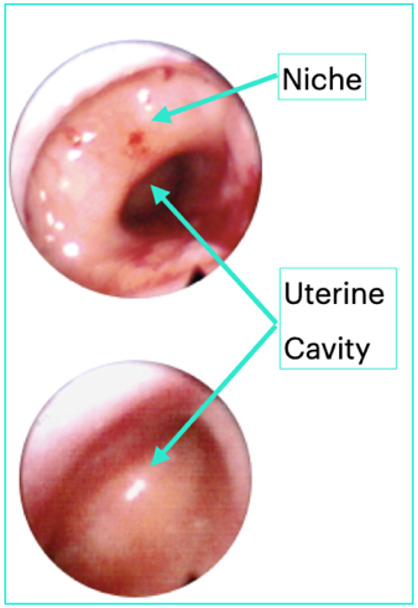
View of a uterine niche compared to the actual cavity at hysteroscopy.

Niche may be seen at the time of hysteroscopy under direct vision, see Figure 5. It has been described as a bulging pouch, sacculation or wedge at the anterior isthmus or cervical canal (Monteagudo et al., 2001; Fabres et al., 2003; Florio et al., 2012). In a prospective cohort study among women undergoing hysteroscopic terilization a niche was detected in 75% of women with a previous CS; however, the niche was defined as any visible defect, disruption or concavity in the anterior uterine wall (Van der Voet et al., 2018). Niches may also be suspected at the time of laparoscopy where previously noted dense adhesions may be apparent from the niche to the anterior abdominal wall (Vervoort et al., 2015).

### Management

Many patients may have a niche detected as an incidental finding. A number of authors have recommended that treatment be reserved for those with symptoms only (Van der Voet et al., 2014b; Demers et al., 2013). For some patients, a conservative approach with a diagnosis that explains their symptoms may be all that is required. To date, the majority of treatment options discussed within the literature have included minimally invasive surgical techniques for treatment.

### Medical treatment

In those patients who have symptoms and are not concerned with immediate fertility, medical therapy with oral contraceptive treatment may be considered. There is inconsistent evidence to support medical treatment and studies have been very small. The aim of medical therapy is to reduce menstrual blood loss and therefore collection of blood in the niche. Thurmond et al. (1999) failed to show any benefit with oral contraceptives while Tahara et al. (2006) in another small study showed the majority of patients (10/11) had a reduction and cessation of bleeding symptoms. In this study, 3 cycles of treatment with a combined pill were given, and after stopping treatment for 3-6 cycles there were no further bleeding symptoms. A further small study compared medical treatment with a combined contraceptive pill versus hysteroscopic resection. This showed a shortening of the number of days of bleeding and an improvement in pain. The resection group had better outcomes overall (Florio et al., 2011). The main side effects reported with medical treatments were breast tenderness and nausea (Tahara et al., 2006; Florio et al., 2011). Anecdotally levonorgestrel intrauterine systems (LNH-IUS) appear to have benefit in some patients. A very small study into the use of a LNG-IUS showed amenorrhoea or cessation of menstrual spotting in the majority of patients (5/6) after 12 months of treatment (Chen et al., 2019). A further study including 40 patients diagnosed with niche treated with a LNG-IUS being compared to hysteroscopic resection showed a significant reduction in post-menstrual spotting with 0 days spotting after 6 months use (He et al., 2020). While we cannot draw any significant conclusions based on these studies, they suggest that that further research would be of benefit to inform clinical practice and patient counselling.

A number of minimally invasive surgical options exist including hysteroscopic resection, laparoscopic repair, combined laparoscopic and hysteroscopic repair and vaginal repair. Hysterectomy is a definitive option for those without future fertility concerns.

### Hysteroscopic niche resection

**Figure 6:**
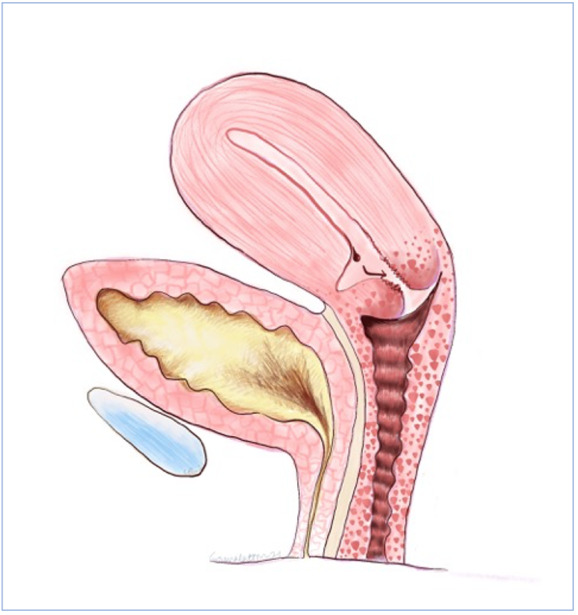
This figure shows how hysteroscopic resection works; by shaving away the distal ridge of the niche to allow drainage of collected menstrual blood.

A systematic review by Van der Voet et al. (2014b) showed success rates of 92-100% with hysteroscopic resection and 100% after laparoscopic and vaginal repair with minimal complications, however it suggested that overall the methodological quality of the studies included was moderate to poor. A multi-centre randomised controlled trial has subsequently been published comparing hysteroscopic niche resection in patients with an RMT ≥3mm to expectant management for 6 months. This showed a reduction in bleeding duration from 7 to 4 days and a reduction in pain symptoms without significant complications (Vervoort et al., 2018 a). Hysteroscopic resection works by shaving away the distal ridge of the niche to allow drainage of collected menstrual blood as shown in Figure 6. Proximal resection may, in theory, increase the risk of cervical incompetence (Vervoort et al., 2018a). Resection of the niche is likely therefore to reduce myometrial thickness. Various thresholds in order to perform the resection have been suggested (between 2.5-4mm of residual myometrium) in order to reduce the risk of bladder injury (Van der Voet et al., 2014b ; Vervoort et al., 2018a ; Li C et al., 2014; Raimondo et al., 2015). A more recent study by Zhu et al. (2020) assessed pre-operative probability of symptom improvement after hysteroscopic niche resection and found that longer pre-operative menstrual duration was associated with improved chance of reduction in menstruation duration by 3 days or more. They also found that a large niche was less likely to result in symptom improvement after hysteroscopic resection. When LNG-IUS was compared with hysteroscopic niche resection, the LNG-IUS appeared to perform better in terms of reduction in post-menstrual spotting and cost- effectiveness after 6 months, although both groups had a significant improvement in symptoms (He et al., 2020). The impact of a hysteroscopic resection on subsequent pregnancy is also unknown and some authors have recommended that it is not performed if future pregnancy is desired (Nezhat et al., 2017, Sipahi et al. 2017).

### Laparoscopic niche repair

**Figure 7:**
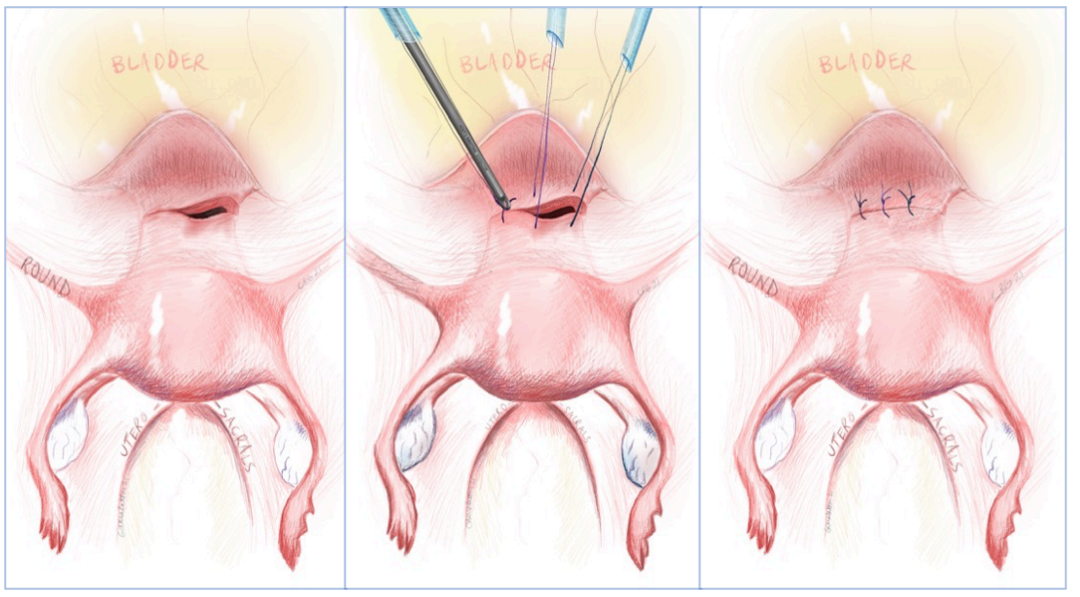
Our technique of laparoscopic niche repair; where first the defect is excised, both angles and the midline of the defect are then secured with extracorporeal sutures.

Laparoscopic niche repair has been demonstrated by many authors including detailed discussion of the technique by Huirne et al. (2017) and in a video publication by Kent et al. (2014). One technique is demonstrated in Figure 7. The largest study to date looking at laparoscopic repair was a prospective cohort of just over 100 patients with an RMT <3mm. The niche was opened with monopolar and excised using cold scissors to encourage wound healing. A double layer repair was performed. In 30% of the women additional shortening of the round ligament was performed to reduce disruptive forces on the uterus. Complications included a case of entry- related vessel injury and conversion to laparotomy, damage to the epigastric vessel requiring suturing in one patient, bladder laceration within one procedure, and uterine perforation. Post-menstrual spotting reduced from 9 days to 2 days, with intermenstrual bleeding reduced from 5 to 0. Dysmenorrhoea and pain with spotting were also reduced. The RMT was increased from 1.2mm to 5.3mm. (Vervoort et al., 2018b). Another, smaller study of 38 patients with RMT <3mm used CO2 laser to excise the defect, again shortening the round ligaments in the case of retroflexed uteri. This study showed that 91% of patients were subsequently symptom free, again with a significant increase in RMT from 1.43 to 9.62mm (Donnez et al., 2017). Combined approaches using hysteroscopy and laparoscopy have been described. The advantage of this approach is to allow adequate bladder reflection, and the hysteroscope can also transilluminate the niche if this is not easily visible (Sipahi et al., 2017). Vaginal approaches have also been described. A study by Zhang (2016) suggested similar outcomes between vaginal and laparoscopic repair.

In terms of fertility and subsequent pregnancy outcomes it is difficult to draw solid conclusions on the benefit to fertility and the risk of pregnancy related complications in the future. A number of studies have suggested good fertility outcomes following hysteroscopic repair (Fabres et al., 2003; Gubbini et al., 2011). A prospective study in 2011 of 34 patients with secondary infertility with other causes excluded showed 100% success within 24 months with around a 10% miscarriage rate. The subsequent 90% delivered via CS (Gubbini et al., 2011). Donnez et al. (2017) reported on some fertility outcomes following laparoscopic repair showing 44% of those with subfertility became pregnant following the repair, with all having CS at 38-39 weeks. The numbers of patients involved in these studies were small and data observational. There is no clear answer in terms of recommended method of repair, and ideally an RCT comparing no treatment with hysteroscopic and laparoscopic repair before firmer recommendations can be made.

## Conclusions

With the UK and worldwide CS rate continuing to rise, long-term gynaecological complications are likely to increase. The caesarean scar niche is now more widely recognised and is thought to develop in up to 50% of those undergoing CS delivery. Its development is likely to be multifactorial and includes low uterine incisions, incomplete closure of the uterine wall (possibly further impacted by locking of sutures), and poor healing especially if dense adhesions develop between the CS scar and the bladder or abdominal wall. The most common presenting symptom is post-menstrual spotting and approximately 65% of niches can be diagnosed using gel hysteroultrasonography. The niche can potentially impact negatively on fertility by creating a hostile environment for a developing embryo but also by preventing sperm transport.

In those women who are asymptomatic, no treatment is necessary. Medical management aims to reduce menstrual blood loss and therefore volume of collected blood, and this may be carried out by oral contraceptive pill or LNG-IUS. Both LNG- IUS, hysteroscopic niche resection and laparoscopic repair improve symptoms, particularly of post- menstrual spotting, and LNG-IUS may be more cost-effective in those not wanting conception. Hysteroscopic niche resection appears to improve both symptoms and implantation rates but caution is needed in those with a thin RMT due to risk of bladder injury. Laparoscopic repair with a large niche and/or thin RMT may be more suitable than a hysteroscopic approach but no data are available to recommend one approach above another. Preterm birth as an outcome after niche repair is also so far unassessed. Hysterectomy may be offered to those whose family is complete, and ideally this should be carried out laparoscopically. Patients should be specifically warned of the risk of damage to the bladder during dissection of the UV fold. In addition to the known potential long-term adverse impacts of CS, the potential to result in cervical niches associated with abnormal uterine bleeding, pain and subfertility need to be considered and patients counselled accordingly.
